# Delirium During Recovery in Patients With Severe COVID-19: Two Case Reports

**DOI:** 10.3389/fmed.2020.573791

**Published:** 2020-10-15

**Authors:** Dong Han, Chenyang Wang, Xiaojing Feng, Jing Wu

**Affiliations:** Department of Anaesthesiology, Union Hospital, Tongji Medical College, Huazhong University of Science and Technology, Wuhan, China

**Keywords:** COVID-19, delirium, neuropsychiatric adverse events, severe patients, case report

## Abstract

Novel coronavirus disease (COVID-19), caused by SARS-CoV-2, has rapidly evolved into a worldwide pandemic, leaving patients with life-threatening respiratory, cardiovascular, and cerebral complications. Here we reported on two patients with severe COVID-19 who experienced delirium in the early stage of recovery and mental illness including fatigue, anxiety, and post-traumatic stress disorder in the post-illness stage of COVID-19. Two patients were admitted to hospital due to clinical symptoms and features of CT and were confirmed for COVID-19 by positive results of a throat swab for SARS-CoV-2. Due to severe respiratory symptoms and a low oxygenation index, they were transferred to the ICU and received invasive mechanical ventilation and sedation. Hyperactive delirium was observed after being transferred out of the ICU. Different treatment measures were taken in time. Delirium did not occur again in hospital, but they showed mental suffering, including fatigue, anxiety, and post-traumatic stress disorder (PTSD), during the 5 month follow-up after discharge.

## Highlights

- These two cases shared a cluster of specific characteristics and risk factors, including the patients being >60 years old, having severe COVID-19, receiving invasive mechanical ventilation and related sedation, high-dose and long-term corticosteroids treatment, and delirium occurring transiently during their recovery period with negative SARS-CoV-2 results and improved laboratory results. These detailed manifestations with dynamic changes in disease and related treatments might provide some clues to clarify the mechanism of psychiatric complications of COVID-19 and further inform targeted interventions.
- Although there was transient delirium during the early phase of recovery, moderate levels of fatigue, anxiety, and PTSD persisted for 5 months after discharge. Long-term follow-up of chronic neuropsychiatric sequelae of SARS-Cov-2 infection is as important as follow-ups on acute neuropsychiatric complications.

## Introduction

Delirium, a disorder characterized by confusion, inattentiveness, disorientation, illusions, agitation, and in some instances autonomic nervous system overactivity, is common in the ICU and exceedingly challenging during the COVID-19 pandemic. However, neuropsychiatric events of COVID-19, including hyperactive and hypoactive delirium, have so far been underreported. Here we reported on two patients with severe COVID-19 who, in the absence of direct brain infection, experienced hyperactive delirium and agitation during the recovery period after being transferred out of the ICU.

## Case 1

**Figure 1:**
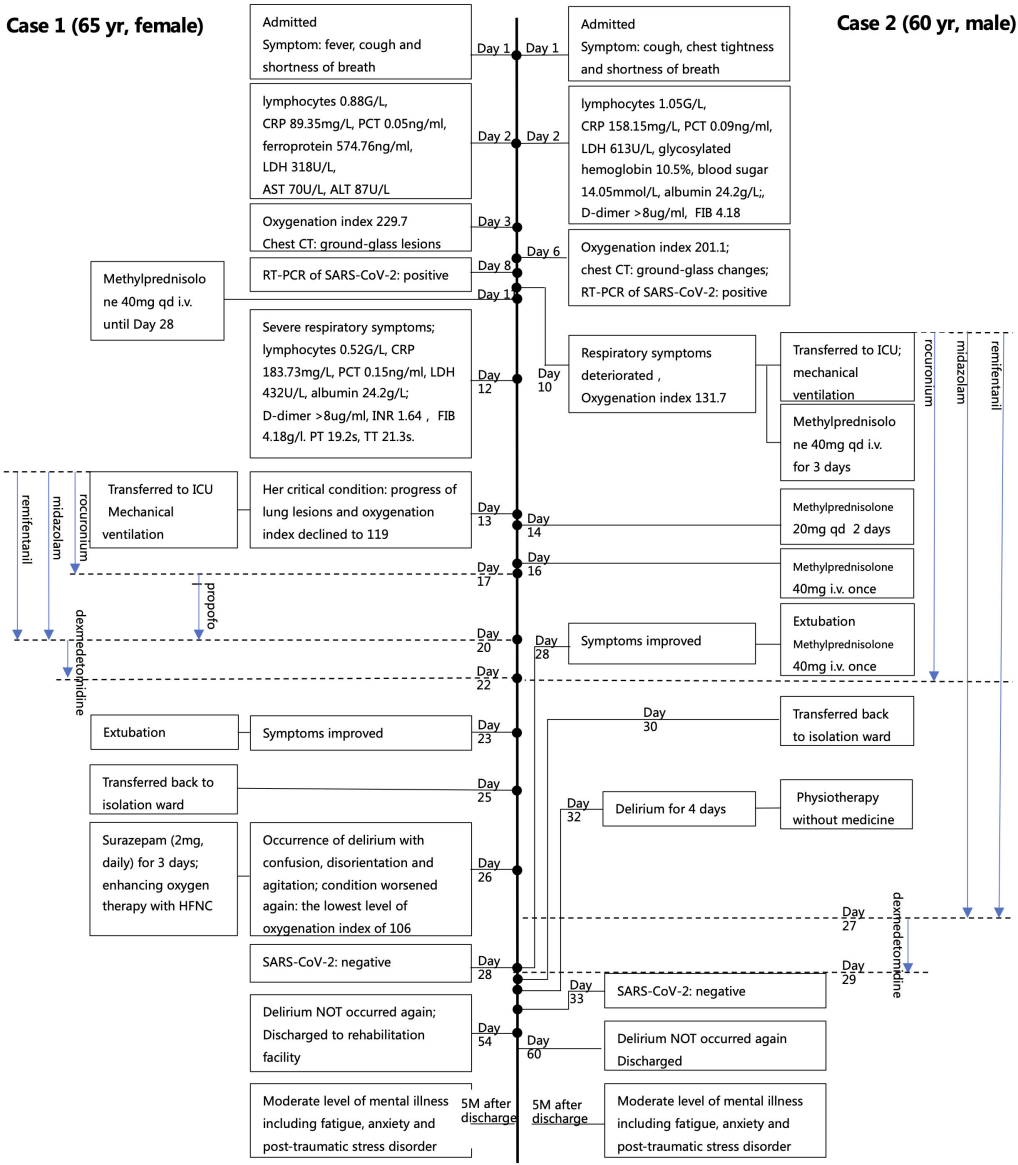
Time-line of main events of two patients. Numbers in the middle line represents the days in hospital. Lymphocytes 1.1–3.2 G/L, CRP 0–8 mg/L, PCT <0.05 ng/ml, Ferroprotein 21.81–274.66 ng/ml, LDH 109–245 U/L, AST 8–40U/L, ALT 5–40 U/L, Albumin 33–55 g/L, Glycosylated hemoglobin 4–6%, Blood sugar 3.9–6.1 mmol/L, D-dimer 0–0.5 ug/ml, PT 11.0–16.0 s, APTT 27.0–45.0 s, INR 0.83–1.36, FIB 2.00–4.00 g/l, TT 14.0–20.0s, Oxygenation index 400–500 mmHg. HFNC, High-flow nasal cannula oxygen therapy.

A 65-year-old female with fever, cough, and shortness of breath on exertion for six days was admitted to hospital. She had normal consciousness and cognition during admission and denied any underlying disease. Abnormal laboratory workup included decreased lymphocytes, elevated inflammatory markers CRP, PCT, and ferroprotein, elevated LDH, and increased markers of liver injury AST and ALT (Figure 1). Arterial blood gas analysis showed a low oxygenation index of 229.7, which suggested acute lung injury (ALI). Chest CT presented with typical ground-glass lesions and diagnosis of COVID-19 was confirmed subsequently by positive results of a throat swab for SARS-CoV-2.

With the diagnosis of severe COVID-19 and hepatic insufficiency, she was transferred to an isolation ward and received support treatments. Unfortunately, her condition continued to deteriorate with severe respiratory symptoms, declining lymphocytes, elevating CRP, PCT, and LDH, emerging hypoalbuminemia, and coagulation dysfunction (Figure 1). Thirteen days after admission, her condition became critical, with the progress of lung lesions in CT, and her oxygenation index declined to 119, which indicated ARDS. She was transferred to the ICU and was given invasive mechanical ventilation immediately. Other treatments, including antivirals, anti-inflammatories, nutritional support, mechanical ventilation-related sedation, and high dose corticosteroids, were given. After 10 days in the ICU, extubation was performed after pulmonary function improved. Two days later, she was transferred back to the isolation ward with improved dyspnea.

At day 2, after leaving the ICU, the patient suddenly developed CNS symptoms, including confusion, disorientation and agitation, without symptoms of Peripheral Nervous System (PNS) and skeletal muscle injury, that met the delirium diagnostic criteria of Confusion Assessment Method for the Intensive Care Unit (CAM-ICU) (1), including an acute onset of mental status changes (Feature 1), attention disorder (Feature 2), and disorganized thinking (Feature 3). Meanwhile, her condition worsened again with respiratory failure at the lowest level of oxygenation index of 106. However, except for IL-6, other laboratory tests indicated improved results and the RT-PCR of the throat swab for SARS-CoV-2 came back negative. Surazepam (2 mg, daily) was given for 3 days with other treatments including oxygen therapy, anti-inflammatories, and anti-infection. Three days later, on day 5 after leaving the ICU, her mental state gradually returned to normal. She was discharged to a rehabilitation facility at Day 29 after leaving the ICU and delirium did not appear again. At a 3 month follow-up after discharge, she expressed experiencing serious fatigue, anxiety, and post-traumatic stress disorder (PTSD). At a 5 month follow-up, her fatigue, anxiety, and PTSD persisted but had improved to a moderate level.

## Case 2

Due to cough, chest tightness, and shortness of breath for 3 days, a 60-year-old male without consciousness or cognitive impairment was admitted to the hospital. His medical history included well-controlled coronary heart disease, poorly-controlled type 2 diabetes with a high glycosylated hemoglobin, and a surgery for cholecystectomy. The blood samples (Figure 1) revealed decreased lymphocytes, elevated inflammatory markers CRP and PCT, elevated LDH, and a high blood sugar level. In addition, hypoalbuminemia and coagulation dysfunction were confirmed according to laboratory tests. Arterial blood gas analysis showed an ALI with a low oxygenation index of 201.1. A chest CT presented with typical ground-glass changes and COVID-19 was confirmed with a positive result of a throat swab for SARS-CoV-2.

The treatment regimen was supplemental oxygen therapy, antiviral treatment, nutritional support, and correcting the coagulopathy. Ten days later, his respiratory symptoms deteriorated significantly with an oxygenation index of 131.7. He was transferred to the ICU and received invasive mechanical ventilation. In addition to strengthening the above treatments, sedatives, corticosteroids, and endotracheal suctioning under fiberoptic bronchoscope was applied with much sputum. At day 18 in the ICU, his lung function improved and extubation was performed. Two days later, he was transferred back to an isolation ward.

At day 3, after being released from the ICU, the patient experienced hyperactive delirium characterized by confusion, disorientation, and agitation with self-extubation of an indwelling gastric tube, without PNS and skeletal muscle injury manifestation. His delirium was measured by the diagnostic criteria of CAM-ICU with an acute onset of mental status changes (Feature 1), inattention (Feature 2), and disorganized thinking (Feature 3). Meanwhile, SARS-CoV-2 turned out to be negative. Physiotherapy was applied, unnecessary psychoactive medication was stopped, and verbal communication (consoling and reminding him of his location and the time) and family presence (his family contacted him via phone or video conversation regularly) was encouraged. Four days later, the delirium completely disappeared. He was discharged at day 30 after leaving the ICU and the delirium did not appeared again. At 3 and 5 month follow-ups after discharge, he still had moderate levels of fatigue and anxiety.

## Discussion

To our knowledge, this is the first detailed report of delirium in two severe COVID-19 patients during the recovery period with negative SARS-CoV-2 results and improved laboratory findings. These two cases shared a cluster of specific characteristics and risk factors, including the patients being >60 years old, having severe COVID-19, receiving invasive mechanical ventilation and related sedation, and high-dose and long-term corticosteroids treatment. Their delirium occurred transiently and did not happen again, however, according to recent follow-ups, moderate levels of fatigue, anxiety, and PTSD have persisted for 5 months after discharge.

Delirium is very common in critical illness, especially in the ICU, with a high prevalence of up to 85% (2). However, the incidence of delirium in COVID-19 is unknown. Mao et al. (3) reported that “impaired consciousness” occurred in only 14.8% of patients with severe COVID-19. A recent study of 58 patients with COVID-19 reported agitation in 40 (69%) patients after the withdrawal of sedation and neuromuscular blockades in the ICU (4). Similarly, agitation, confusion, and hallucinations occurred in 61% of patients in the acute stage of SARS (5). Underreporting of delirium may be common because it is hard to screen delirium and other mental illness under a heavy workload and epidemiologic precautions during this pandemic (6). In the post-illness stage of SARS and MERS, persistent psychiatric impairment, including depression, anxiety, post-traumatic stress disorder, and fatigue, are rather common, but there is little data yet on COVID-19 (7). More attention should be paid to the long-term psychological prognosis related to COVID-19 recovery.

Potential mechanisms of delirium in COVID-19 might be heterogeneous and multifactorial, including direct effects of the virus infiltration, hypoxia, sepsis, and/or the subsequent host immunologic response, medical interventions, and so on. Direct CNS invasion by the virus appears to occur rarely and might be not the related factor in these two cases because of the absence of manifestations of brain infection. Our guess may be further supported by the negative SARS-CoV-2 results and improved laboratory results during delirium. Although the patients experienced severe hypoxia and high inflammatory reactions complicated with coagulation dysfunctions, hepatic insufficiency, and heart failure, delirium occurred after extubation and leaving the ICU. Correspondingly, we speculated that the possible mechanism of delirium in these two cases might not be the direct effect of viral infection, but might be a post-infection immunologic response and immunomodulatory treatment (corticosteroid therapy). In addition, persistant invasive mechanic ventilation and related sedation might also take part in and/ or aggravate the neuropsychiatric complications. It is reported that steroid-induced psychotic disorder occured in 13 (0.7%) of 1,744 patients with SARS in the acute stage (8). In addition, the environment factors caused by being isolated from family and limited support from healthcare workers contributed to the occurrence of delirium. Thus, from the two cases, more exploration on psychoneuroimmunology mechanisms, including the characterization of immune host responses, exploration of genetic associations, and comparison with different medical interventions, especially immunomodulatory treatments, might be useful.

Although data about the acute effects of the illness on neuropsychiatric complications are limited, the evidence from SARS and MERS suggested the high mortality might be linked with poor prognosis of psychosis (9, 10). So far, no medical intervention can be routinely recommended to apply for prevention and management (11, 12). Non-pharmacological interventions, such as comfort and regular orientation from family, friends, and healthcare workers, have proven to be safe and effective methods for treating delirium (13, 14).

There are several limitations in our case reports. First, during the period of the COVID-19 outbreak, in order to avoid cross-infection and reduce the burden on front-line health workers, advanced neuroimaging techniques such as CT and MRI and diagnostic procedures were purposefully avoided, which is necessary to elucidate the causality and etiopathogenic mechanisms. Second, we here reported about delirium in only two patients and obtained information on a recent follow-up period of 5 months after discharge, which is not enough to evaluate the neuropsychiatric impact of COVID-19. Population-based multi-center research about delirium and longitudinal monitoring of neuropsychiatric complications of COVID-19 is still needed.

In summary, we reported on two severe COVID-19 patients who experienced delirium in hospital and persistent psychiatric impairment after discharge. Those detailed manifestations during the dynamic changes of disease and related treatments might provide some clues to clarify the psychoneuroimmunological mechanism of psychiatric complications of COVID-19 and further contribute toward targeted interventions. Long-term follow-ups of chronic neuropsychiatric sequelae of SARS-Cov-2 infection is as important as follow-ups of acute neuropsychiatric complication.

## Ethics Statement

Ethical approval not required according to local legislation and national guidelines. The patients/participants provided their written informed consent to participate in this study. Written informed consent was obtained from the individual(s) for the publication of any potentially identifiable images or data included in this article.

## Author Contributions

DH: acquired, analyzed patient data and drafted the manuscript. CW: acquired, analyzed the data and revised the manuscript. XF: revised the manuscript. JW: design and conceptualized the report, interpreted clinical data, and revised manuscript. All authors contributed to the article and approved the submitted version.

## Conflict of Interest

The authors declare that the research was conducted in the absence of any commercial or financial relationships that could be construed as a potential conflict of interest.
